# Three-dimensional assessment of the mandibular lingual foramina with implications for surgical and implant therapy: A multicentre cross-sectional study

**DOI:** 10.1016/j.jobcr.2023.01.002

**Published:** 2023-01-06

**Authors:** Ninad Milind Padhye, Vinayak Umesh Shirsekar, Rukhshanda Siraj Rakhangi, Paul Mathai Chalakuzhy, Akshada Vinayak Joshi

**Affiliations:** a Ceramco Dental Care, Lokhandwala Complex, Andheri, Mumbai, India; b Mahatma Gandhi Vidyamandir Dental College, Nashik, Maharashtra, India; c Nair Hospital Dental College, Mumbai, Maharashtra, India; d Mahatma Gandhi Missions' Dental College, Navi Mumbai, Maharashtra, India

**Keywords:** Anatomy, Cone beam computed tomography, Dental implant, Mandible

## Abstract

Treatment planning for dental implants in the anterior mandible is often complicated by the presence of vascular structures. The aim of our study was to investigate the prevalence, location and morphology of the mandibular lingual foramen (LF) through cone beam computed tomography (CBCT) scans and contribute to its anatomical knowledge in an Indian population. A total of 400 mandibular anterior CBCT scans from 4 centers were included in this retrospective analysis. The vertical distance from alveolar crest (H_cre_) and inferior border of mandible (H_inf_) to the LF, horizontal distance from lingual canal to labial cortical plate (LC-CP), length of the lingual canal (LLC) and diameter of the LF were measured. Data was analysed using Wilcoxon signed rank test and compared between median and lateral LF. 14 (3.5%) scans were excluded due to non-visualization of LF. A lateral LF was detected in 149 scans (38.6%), predominantly in the canine region (61.7%). H_cre_ was significantly higher for median LF (16.35 ± 4.59 mm) than lateral LF (12.94 ± 3.92 mm) (*p* < 0.001), while H_inf_ did not show significant difference between median (11.38 ± 3.62 mm) and lateral (12.94 ± 3.92 mm) LF (*p* = 0.0032). The LC-CP, LLC and diameter of LF averaged at 5.05 ± 1.76 mm, 6.26 ± 1.82 mm and 0.88 ± 0.72 mm respectively. The LF can be visualized in CBCT scans with a prevalence of 96.5%. This study stresses on the need for a CBCT, prior to surgeries in anterior mandible to avoid excessive bleeding episodes.

## Graphical abstract

**Figure:**
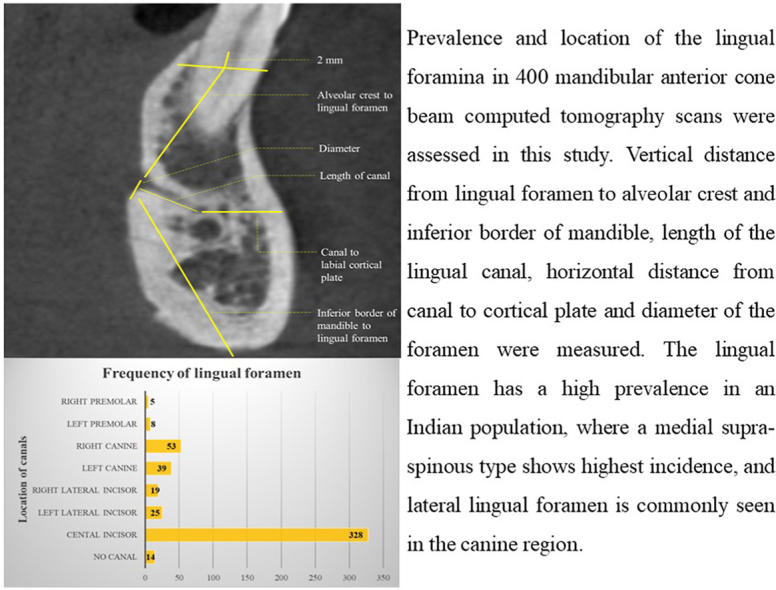

## 1 Introduction

Over the past 3 decades, dental implants have shown a tremendous gain in popularity for rehabilitation of missing teeth. Endosseous implants can predictably replace missing teeth with a survival rate above 90% over a 5-, 10-, and 13-year period.^1^^,^^2^ The mandibular incisors are the most commonly lost teeth due to periodontal disease, thus making anterior mandible an area of particular interest in implant therapy.^3^ The mandibular anterior bone also serves as an optimal region for implant placement to support overdentures.^4^ Furthermore, autogenous grafts for ridge augmentation procedures are often harvested as blocks from the mandibular symphysial region.^5^ Considering the importance of the mandibular anterior region, a detailed knowledge about the anatomical landmarks is essential to avoid surgical complications.

The lingual foramen (LF) is a major anatomical structure in the anterior mandible. It serves as an exit for the artery developed from the anastomosis of the two sublingual arteries, and acts as a nutrient canal.^6^^,^^7^ It may be located either superior or inferior to the genial tubercle, and is usually present along the midline of the internal region of the mandibular symphysis.^8^^,^^9^ A close relationship has been suggested between mandibular LF and bleeding risk during surgery.^10^ Due to its proximity to the sublingual space, damage to this structure could potentially be fatal.^11^

The cone beam computed tomography (CBCT) is an excellent tool to reduce inferior alveolar nerve impingement in implant dentistry.^12^ While it may be impossible to detect the LF on two-dimensional panoramic radiographs, it can be visualized on CBCT from multiple perspectives. Few studies have evaluated the anatomical structures of the anterior mandible in Brazilian, Chinese and Iranian populations.^13^, ^14^, ^15^ With an annual approximate of 14 million implants placed globally, India contributes to 4% of the share.^16^^,^^17^ Hence, it is imperative to understand the craniofacial anatomy, along with its variations, among Indian population, to prevent surgical complications. Hence, the object of our study was to contribute to the anatomical knowledge of the LF by assessing it three-dimensionally using CBCT. In this way, we hope to help dental surgeons and oral implantologists with the treatment planning in the anterior mandibular region.

## 2 Materials and method

This retrospective study included CBCT images taken from 4 centers across India using the same scanning machine (Galileos®, Sirona, Bensheim, Germany). The patient positioning was standardized with set exposure factors as advocated by the manufacturer (0.4 mm Voxel, 120 kVp, 3–8 mA). Ethical clearance was obtained (ISBEC/ NR-21/ KM-VM/ 2019) and subjects without radiographic or clinical evidence of periapical lesion, periodontal disease, history of trauma or fracture in anterior mandible were recruited. Exclusion criteria were subjects who had received any kind of surgical treatment, including regenerative procedures, in the anterior mandible. Completely edentulous mandibles were excluded due to no tooth landmark for positioning the structures.

**Fig. 1:**
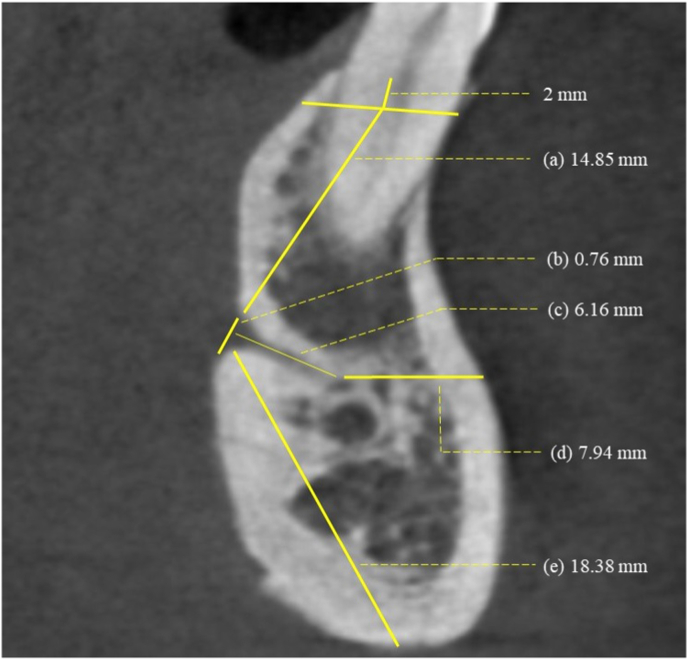
Measurements in the coronal section of the cone beam computed tomography (CBCT) scan. a. Vertical distance from 2 mm apical to the alveolar crest to the LF (Hcre). b. Diameter of the LF. c. Length of the lingual canal (LLC). d. Horizontal distance from the anterior-most point of the lingual canal to the labial cortical plate (LC-CP). e. Vertical distance from the inferior border of the mandible to the LF (Hinf).

The same computer screen was used to present all the CBCT images, and the same software was used to analyse the scans (Galaxis®, Sirona Dental Systems, Bensheim, Germany). All possible setting options could be adjusted (contrast, scroll volume), and all the scans were visualized in full volume size. The measurements were performed by a single examiner (NP) and later verified by a second examiner (VS). A high level of agreement was noted for the inter-examiner calibration (K > 0.80). In a sagittal section, a thin canal-like radiolucency originating from the lingual surface of the mandible was identified as the lingual canal, and its opening was considered to be the LF. The following measurements were obtained for the mandibular anterior region (Fig. 1):

1) Vertical distance from 2 mm apical to the alveolar crest to the LF (H_cre_), 2) Vertical distance from the inferior border of the mandible to the LF (H_inf_), 3) Horizontal distance from the anterior-most point of the lingual canal to the labial cortical plate (LC-CP), 4) Length of the lingual canal (LLC), 5) Diameter of the LF, 6) Position of the LF relative to the genial tubercle.

Statistical analysis was performed using “MedCalc Statistical Software” version 13.3.1 (MedCalc Software bvba, Ostend, Belgium; http://www.medcalc.org; 2014). Descriptive analysis of all data was done including the mean and standard deviation at alpha 0.05 (95% confidence intervals). Intra-subject discrepancies for the median and lateral foramina were detected using the Wilcoxon signed rank test for paired data.

## 3 Results

**Fig. 2:**
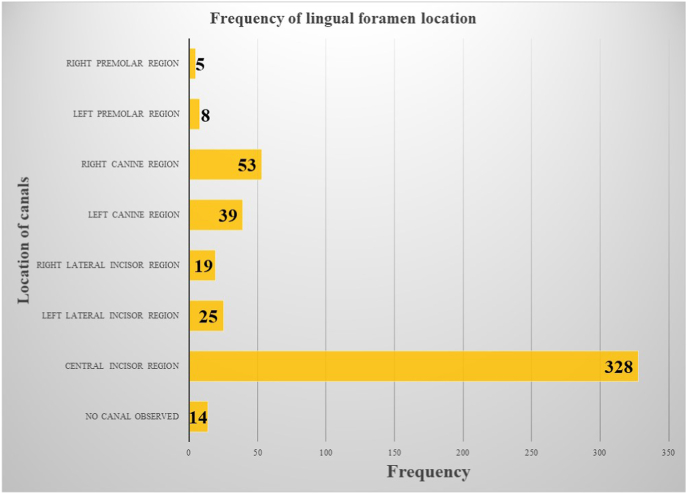
Frequency of lingual foramen in various areas of the mandibular symphysis among the scans assessed in our study.

A total of 400 subjects who visited the 4 CBCT centers across India from July 2020 to March 2021 were included in this retrospective evaluation. Among the study participants, 295 were males (73.75%) and 105 were females (26.25%) with a mean age of 41.5 years (range – 22 to 76 years). Out of the 400 scans, 14 (3.5%) were excluded due to non-visualization of the LF in the coronal sections. Thus, a total of 386 CBCT scans were included in this study. A median LF (apical to central incisors) was detected in 328 scans (84.97%), while a lateral LF was detected in 149 scans (38.6%) (Fig. 2). Among the lateral LF, 92 (61.7%) were observed in the canine region.

The mean H_cre_ and H_inf_ values were 15.01 mm ± 5.67 mm and 11.76 mm ± 3.3 mm respectively. The H_cre_ for the median LF was significantly greater (16.35 mm ± 4.59 mm) than the lateral LF (12.94 mm ± 3.92 mm) (*p* = 0.0032). Although the H_inf_ value for the lateral LF (12.54 mm ± 4.13 mm) was greater than the medial LF (11.38 mm ± 3.62 mm), there was no statistically significant difference (*p* = 0.096).

**Fig. 3:**
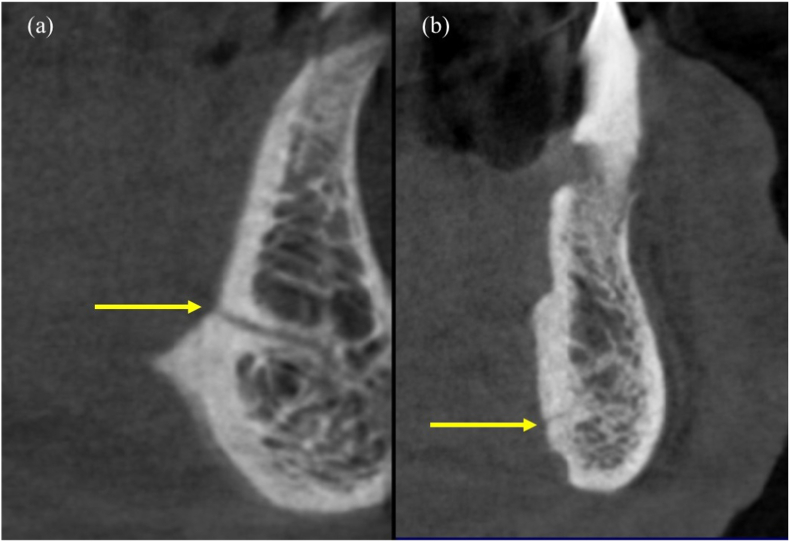
Lingual foramen positioned (a) superior and (b) inferior to the genial tubercle.

**Fig. 4:**
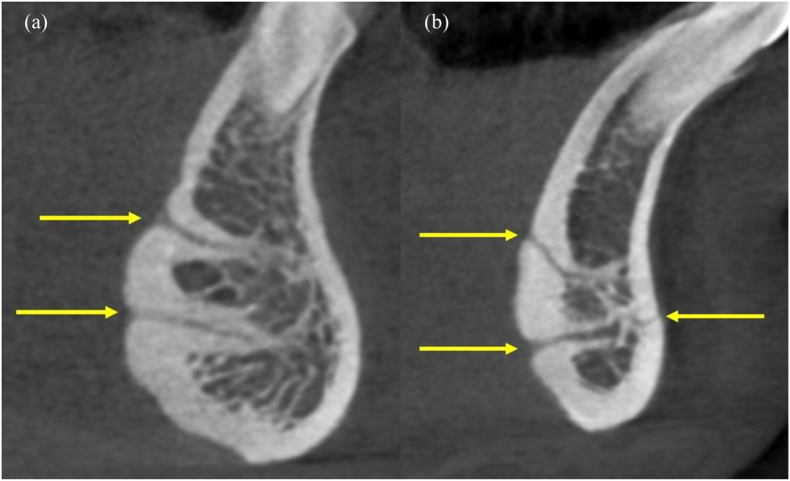
(a) Foramina openings both superior and inferior to the genial tubercle; (b) Foramina openings both superior and inferior to the genial tubercle, with a labial foramen opening.

The LC-CP averaged at 5.05 mm ± 1.76 mm, while the LLC averaged at 6.26 mm ± 1.82 mm. The diameter of the LF ranged from 0.3 mm to 1.83 mm and averaged at 0.88 mm ± 0.72 mm. The LC-CP, LLC and diameter values did not show a significant difference between the median and lateral LF. Among the included scans, 279 (72.27%) and 138 (35.75%) showed the LF to be positioned superior and inferior to the genial tubercle respectively (Fig. 3), while 31 (8.03%) of the scans displayed foramina openings both superior and inferior to the genial tubercle (Fig. 4a and b). A labial canal opening was seen in 8 scans (2.07%) (Fig. 4b).

## 4 Discussion

This retrospective cross-sectional study analysed the presence and location of the mandibular LF, with the objective of recognizing and avoiding surgical complications, particularly during dental implant procedures. The study data was procured from multiple CBCT centers across India, and thus provided an overview of the anatomical structures of anterior mandible in an Indian population.

A plethora of evidence suggests the accuracy and reliability of CBCT for pre-operative implant site assessment and treatment planning.^18^, ^19^, ^20^ It is particularly useful to determine the alveolar bone topography, localization of vital anatomical structures, identification of possible pathological findings and fabrication of implant surgical guides. Moreover, the CBCT has shown to have a relative dimensional error of less than 1% when compared with digital calliper measurements.^21^ This suggests the precision of CBCT for linear measurements and assessing anatomical aberrations. Thus, in a consensus report, the International Congress of Oral Implantology recommended that CBCT may be considered as a reliable option when conventional radiography fails to accurately assess the three-dimensional anatomical presentation.^22^

The median LF was reported to have a prevalence of 84.97% among the analysed scans, while the lateral LF had a prevalence of 38.6%. This was in agreement to a previous study by Xie et al. in a Chinese population (90.9% for median lingual foramen).^14^ On the contrary, prevalence of the lateral LF was considerably higher in Indians as compared to Turkish population (21–24%),^23^^,^^24^ while significantly lower as compared to Japanese (80%)^25^ and German population (52.5%).^26^ However, along with inter-racial osteological differences, these variations in the frequency may even be attributed to diverse equipment, settings and voxel size of the CBCT scans used in these studies. Often, small dimensional anatomic structures are masked when the slice of the CBCT scan is thick.^27^ This was seen in a study by Scaravilli et al., who did not report any lateral LF with 1.5 mm thickness CBCT slices.^28^ Furthermore, in CBCT scans with a lower resolution setting, the LF, that averaged at 0.88 mm diameter, may not be visualized due to a lack of reformatted slice in the cross-section exactly around the foramina.^14^ In a few scans included in our study, the LF presence could not be confirmed, and such foramina were acknowledged as non-existent. Thus, the prevalence of the lateral LF might have been underestimated than reported in our study.

The H_cre_ distance was measured from 2 mm apical to the alveolar crest. This was significant from a clinical point of view while placing implants. The mean H_cre_ in our study was 15.01 mm ± 5.67 mm, ranging between 3 mm and 28 mm. In contrast to a study by Yildirim et al., our analysis showed a significant difference in the vertical alveolar bone height between the median and lateral LF.^19^ Most of the assessed scans showed a supra-spinous LF opening (72.27%). The H_inf_ value was also higher in these samples compared to the infra-spinous LF opening. However, the H_cre_ value was relatively higher when the foramen opening was infra-spinous. This suggests a greater height of available alveolar bone for dental implants in subjects with LF positioned inferior to the genial tubercle. Two lingual canal openings were noted in about 8% of our samples, with similar incidence to that of Chinese population (12%).^14^ The available bone height for implant placement was considerably lesser in these subjects, due to the superior positioning of the canal.

The mandibular canine region is the most common location of severe haemorrhage during surgical procedures.^28^, ^29^, ^30^ A higher prevalence of the lateral LF in this region, according to our study and also previous literature by Wang et al., may explain the high rate of bleeding episodes.^31^ Also, the mandibular symphysis is a popular region for harvesting autogenous bone graft, particularly cylindrical trephine cores and block grafts. The average LC-CP value of 5.05 mm ± 1.76 mm suggests that a graft of thickness greater than 4 mm obtained from such sites may result in moderate haemorrhage. This is particularly significant in subjects with a lateral lingual canal, as block grafts are usually harvested from the mandibular lateral incisor-canine region.^5^

The diameter of the LF ranged from 0.3 mm to 1.83 mm. According to the diameter, we classified the foramina into >1 mm and ≤1 mm. It was found that 75.1% of the subjects had LF ≤ 1 mm. This suggests a relatively lower risk of severe haemorrhage in these subjects.^32^

To prevent complications, the operator must also be familiar with the contours of the mandible along with its arterial supplies. Careful implant angulation with appropriate length must be chosen to prevent encroachment into the sublingual and digastric fossa of the anterior mandible.^33^ With advanced age and alveolar bone resorption, a relative superficialisation of the vascular network is noted in the mandible.^34^ Since mandibular resorption was unclassified in our study, it can be assumed that in atrophic ridges, the LF are even more superficially located. Future studies could assess and categorize the LF according to the degree of alveolar resorption, and compare edentulous versus dentulous mandibles.

## 5 Conclusion

The present study showed a high prevalence of the LF in the Indian population. A median LF with supra-spinous opening showed the highest prevalence, while the lateral LF were commonly detected in canine region. To avoid unwanted bleeding during implant surgery, the mandibular LF must be identified in the CBCT scans as a part of routine diagnostic procedure to determine the surgical safe-zone in anterior mandible.

## Sources of funding

Nil.

## Declaration of competing interest

Nil.
